# The complete chloroplast genome sequence of *Myricaria elegans*: an endemic species to the Himalayas

**DOI:** 10.1080/23802359.2021.1997111

**Published:** 2021-11-03

**Authors:** Mei Han, Mingyue Xu, Shizhen Wang, Liangdan Wu, Yuan Shi, Tao Su

**Affiliations:** a Co-Innovation Center for Sustainable Forestry in Southern China, College of Biology and the Environment, Nanjing Forestry University, Nanjing, China; b Key Laboratory of State Forestry Administration on Subtropical Forest Biodiversity Conservation, Nanjing Forestry University, Nanjing, China

**Keywords:** *Myricaria elegans*, *Myricaria*, complete chloroplast genome, phylogenetic analysis

## Abstract

*Myricaria elegans*, an endemic species to the Himalayas, is a distinctive deciduous shrubbery plant-primarily distributed in the Qinghai-Tibet Plateau and adjacent regions in China. It is a kind of fuelwood, medicinal, and ecology-protecting woody plant species. In this study, the whole chloroplast (cp) genome sequence of *M. elegans* was assembled and characterized by high-throughput sequencing data. The complete cp genome of *M. elegans* was 155,245 bp in length with a GC content of 37.4%. It contained a large single-copy region (LSC) of 84,846 bp, and a small single-copy region (SSC) of 18,290 bp, which were separated by a pair of 26,053 bp inverted repeat regions (IRs). The cp genome of *M. elegans* was composed of 130 genes, including 85 protein-coding genes, 37 transfer RNA (tRNA) genes, and eight ribosomal RNA (rRNA) genes. Phylogenetic analysis revealed that *M. elegans* formed a clade with *Myricaria*, and it showed a close relationship with *Myricaria prostrata*.

*Myricaria elegans* Royle. (Tamariscineae), a shrubbery woody species with reddish brown-black bark is endemic to the Himalayas (Zhang et al. 2006, 2014). It is mainly distributed within the Qinghai-Tibetan Plateau and the adjacent Xinjiang region in China (Liu et al. 2009). *M. elegans* is a kind of fuelwood, ecology-protecting plant, and medicinal xylophyta (Khan et al. 2010). It has been used by Tibetan people from ancient times for treating bruises, wounds, and burns. Same to its isogeneric plant *Myricaria germanica*, being a source of Tibetan medicine “Om-bu”, *M. elegans* has the effects of expelling wind to resolve exterior syndrome, clearing heat and removing toxic substances, activating the collaterals, promoting eruption and relieving cough (Li et al. 2015; Dawa et al. 2021). In addition, *M. elegans* is enriched in pentacyclic triterpenes, phenols, flavonoids, and other bioactive compounds, which makes it a conspicuous antibacterial and anti-inflammatory medicinal material (Khan et al. 2010). Despite its versatile potentialities, this species remains overlooked and underutilized. So far, a few scientific studies have been conducted on it; resultantly our knowledge of its physiological, molecular characters and pharmaceutical activities is minimal. Of note, owing to its equivocal morphology and property of liable to cross, the taxonomy and systematics position of *M. elegans* have long remained ambiguous. Some researchers considered it to be a unique intermediate of *Tamarix* and *Myricaria* (Qaiser and Ali, 1978). But others proposed to keep *M. elegans* in its monotypic genus in the name of *Myrtama* (Zhang et al. 2006). To get an insight into the molecular basis on the taxonomy of *M. elegans*, in this study, the complete plastid genome of *M. elegans* was determined. Our result provides valuable information on phylogenetic, population, conservation, and application aspects of this neglected woody species.

The fresh leaves of *M. elegans* were sampled from Gar County, Ngari Prefecture, Tibet, China (32°04′ N, 80°36′ E). The voucher specimen (ID: ST20210502002) was deposited in the herbarium of Nanjing Forestry University (NJFU), China (Tao Su, sutao@njfu.edu.cn). The total genomic DNA was extracted by using the DNeasy Plant Mini Kit (Qiagen, Valencia, CA, USA). The whole-genome sequencing was implemented on a Novaseq platform (Illumina, San Diego, CA) following the manufacturer’s protocol. In total, about 1.97 GB of raw data were obtained and used to *de novo* construct the complete cp genome. After quality assessment and filtering by the fastp program (Chen et al. 2018), the clean data was assembled using the program GetOrganelle (Jin et al. 2020). The resulting contigs were linked based on overlapping regions of the reference chloroplast genome sequence of *Myricaria prostrata* (GenBank accession no.: NC_046761). Gene annotation was performed on the Geseq database (Tillich et al. 2017) combined with BLAST searches.

The complete chloroplast/plastid genome sequence of *M. elegans* and gene annotations were submitted to GenBank under the accession number MZ489116. The cp genome of this species was determined to comprise double-stranded, circular DNA of 155,245 bp in size, including two inverted repeats (IR) regions of 26,053 bp each, separated by a large single-copy (LSC) and a small single-copy (SSC) region of 84,846 and 18,290 bp, respectively. The genome contained 130 genes, including 85 protein-coding genes, 37 transfer RNA (tRNA) genes, and eight ribosomal RNA (rRNA) genes. The overall GC content is 37.4%.

The sequence alignment of *M. elegans* with 18 representative species from the Caryophyllales order and Salicales family (outgroups) was performed by using MAFFT v7.307 (Katoh and Standley 2013). The maximum-likelihood (ML) phylogenetic tree was constructed by FastTree version 2.1.10 (Price et al. 2010). The results showed that *M. elegans* formed a clade within *Myricaria*, and it was closely related to *M. prostrata* (Figure 1).

**Figure 1.**
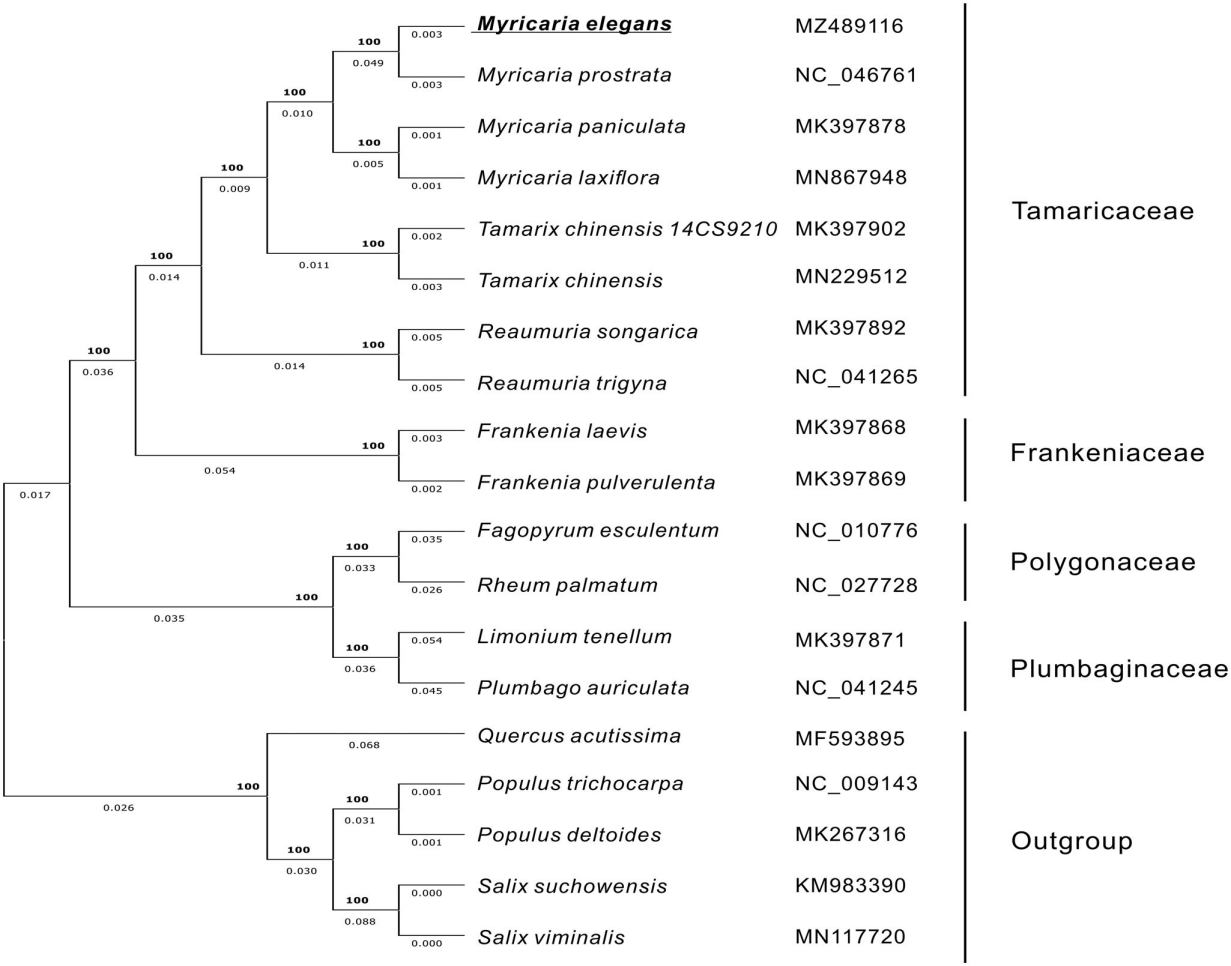
The Maximum-likelihood (ML) phylogenetic tree of *Myricaria elegans* and 18 relative species was constructed based on complete chloroplast genome sequences. The GenBank accession number for each species is listed after the scientific name. The bootstrap support value is labeled for each node.

## Data Availability

The complete plastid genome data that support the findings of this study are openly available in GenBank of NCBI (https://www.ncbi.nlm.nih.gov/) under the accession no. MZ489116. The associated BioProject, SRA, and Bio-Sample numbers are PRJNA749679, SRR15255748, and SAMN20397105, respectively.
